# Pain Cured by Hypodermic Injections of Cold Water

**Published:** 1881-11

**Authors:** 


### PAIN CURED BY HYPODERMIC INJECTIONS OF COLD WATER.

In the *Gaceta Medica* of Venezuela, Dr. Ponte relates his experiences in several instances in which he employed water hyperdermically for the relief of pain. The first case was that of a boy, who was suffering from an attack of intercostal neuralgia, so severe as almost to endanger the life of the patient by interference with respiration. Not having any morphine with him, the author determined to work upon the imagination of the sufferer by injecting pure cold water over the location of the pain, a procedure which, much to his astonishment, was followed by permanent relief. Impressed with this fact, Dr. Ponte resolved upon further experiments. The next case was one of toothache. In order to eliminate the imaginative element, he informed the patient of the treatment to be employed, for the execution of which permission was rather reluctantly given. An injection practised upon the side of the face nearest to the pain was followed by considerable ardor, but in less than a minute the odontalgia had subsided. Animated by these results, he employed cold water injections in a variety of different pains, always with happy issue, even in cases where morphine had been the drug previously administered. Another patient had been suffering nine years from intense gastro-intestinal neuralgia, which baffled all remedies. The pain came on after meals, and its violence was such as to cause her frequently to faint. When first seen by the writer, she was utterly prostrated. Two injections relieved the pain, and subsequent tonic treatment restored her to perfect health. Several hundred cases have been treated in the manner described, with good results. No explanation is given as to the action of the remedy.—*N. Y. Med. Record.*

